# A Framework to Improve Energy Efficient Behaviour at Home through Activity and Context Monitoring

**DOI:** 10.3390/s17081749

**Published:** 2017-07-31

**Authors:** Óscar García, Javier Prieto, Ricardo S. Alonso, Juan M. Corchado

**Affiliations:** University of Salamanca, BISITE Research Group, Edificio I+D+I, 37007 Salamanca, Spain; javierp@usal.es (J.P.); ralorin@usal.es (R.S.A.); corchado@usal.es (J.M.C.)

**Keywords:** real-time localization system, wireless sensor networks, energy behaviour, energy savings, social computing, recommendation system, virtual organization of agents

## Abstract

Real-time Localization Systems have been postulated as one of the most appropriated technologies for the development of applications that provide customized services. These systems provide us with the ability to locate and trace users and, among other features, they help identify behavioural patterns and habits. Moreover, the implementation of policies that will foster energy saving in homes is a complex task that involves the use of this type of systems. Although there are multiple proposals in this area, the implementation of frameworks that combine technologies and use Social Computing to influence user behaviour have not yet reached any significant savings in terms of energy. In this work, the CAFCLA framework (Context-Aware Framework for Collaborative Learning Applications) is used to develop a recommendation system for home users. The proposed system integrates a Real-Time Localization System and Wireless Sensor Networks, making it possible to develop applications that work under the umbrella of Social Computing. The implementation of an experimental use case aided efficient energy use, achieving savings of 17%. Moreover, the conducted case study pointed to the possibility of attaining good energy consumption habits in the long term. This can be done thanks to the system’s real time and historical localization, tracking and contextual data, based on which customized recommendations are generated.

## 1. Introduction

During the last decades, the efficient use of energy resources has been established as one of the most concerning problems for our society and more resources are being allocated for its resolution [1]. For the European Union (EU), energy efficiency is a key strategy for the sustainable growth of our planet, thus it set a clear objective: in 2020, the consumption of traditional energy and greenhouse emissions should be reduced by 20% [2]. Within this broad framework, energy saving and energy efficiency policies must be applied at all levels and sectors, to achieve a global awareness of the problem. Further, the optimization of energy resources has to be considered for all uses, regardless of the punctual impact of each action on the total energy consumption.

One of the sectors in which proposals for the optimization of energy consumption have a wide impact are households: in 2015, this sector consumed 33% of total energy [2]. The EU recognized this issue and determined around 650 action measures in the residential sector. Some of these measures included the use of smart meters, information campaigns or the encouragement to change user behaviour [1]. Currently, household energy consumption within the EU is around 25% of total consumption and, although around 1.8% annual reduction in consumption has been achieved since 2000 in this sector [2], there is still a lot of room for improvement. Much of this decline in consumption has been driven by both technical improvements in devices and by the increase in the price of energy, which is why the effectiveness of policies that seek to influence and change consumer’s behaviour should not be overlooked [1].

Numerous studies have been conducted to measure the impact of home occupants’ behaviour on energy consumption [3,4,5,6,7]. The determination of behaviour patterns, which provide feedback and guidelines to users, is one of the most widespread topics [8]. Two main lines of work are well differentiated within this area: on the one hand, works focused on the simulation of behaviour to obtain patterns [6,7,8,9], and, on the other hand, the works that use real data for gathering behaviour patterns [5,10,11,12]. The former does not obtain good results since simulations do not consider spontaneous and unplanned modifications in environmental conditions. However, systems that use real-time data, either from user tracking or contextual information, even price of energy, make these data available to users, allowing for a substantial improvement in the effectiveness of the designed solutions [13,14,15,16,17,18,19].

Technology therefore plays an essential role in the acquiring of real-time information that surrounds the residents at all times. The design, deployment and use of context-aware systems allows determining what factors relate to energy consumption at a given time, including the location of the occupants, the use of domestic appliances or devices (open window, light on, TV off, etc.) or environmental conditions (temperature, humidity, time, lighting, etc.). Wireless Sensor Networks (WSN) and Real-Time Localization Systems (RTLS) become powerful tools that collect essential data in this kind of solutions [20,21,22,23].

Some of the most common solutions in this area are focused on the search for patterns in the use of lighting, windows, blinds or heating [3,15]. Some works go further and use the position of users to determine richer and more accurate behaviour patterns [16,18]. Moreover, the implantation of smart meters in homes is becoming universal [16,24]. These devices allow determining energy consumption at home in real-time, which means that they are able to offer extensive feedback to users, while other solutions give recommendations to users to help them reduce consumption on the basis of historical data and not by considering current activity [24].

Furthermore, the penetration of social networks in our daily life has opened up new possibilities of improving energy consumption by influencing people’s behaviour [25]. In this sense, Social Computing is an area of computer science focusing on collective and collaborative actions [26]. Thus, some solutions use social tools to foster the use of energy saving systems [27,28] and others use social information to aid decision-making or to share energy saving information [29,30].

Despite the examples given above, very few works combine the use of Wireless Sensor Networks and indoor positioning systems for the collection of data in real-time and Social Computing. Moreover, they do not cover the problem in a global way, which means that they do not consider all the aspects that influence the behaviour of home residents. Finally, this paper analyses several experimental systems designed to improve energy consumption. All of them attain savings of between 10% and 17%, which is still far from the EU 20% objective.

This work presents the CAFCLA framework (Context-Aware Framework for Collaborative Learning Applications) which combines WSN and Social Computing to develop activities for users rapidly and effectively, helping them to change their energy consumption habits. The framework, designed from the perspective of Social Computing, is used as the basis for the implementation of applications that include context-aware, localization, and social functionalities.

To validate its conceptualization and design, the framework is assessed by an experimental use case, which emphasizes the effectiveness of using Real-Time Localization Systems and Wireless Sensor Networks. These systems are used for the development of a social machine (recommendation system) that allows improving energy saving at homes, as well as to positively influence the behaviour of users, thanks to the monitoring of their activities and their tracking.

The rest of the paper is organized as follows: Section 2 describes previous works focusing on context awareness for energy monitoring, RTLS for energy saving, and Social Computing for energy efficiency. After this, Section 3 presents CAFCLA and how it is used to achieve the objectives of the work: Section 3.1 describes the problem and the framework, Section 3.2 approaches the RTLS, Section 3.3 depicts how location is enhanced and how it is integrated within CAFCLA, and Section 3.4 provides information on Social Computing recommendation systems that make use of this framework for their implementation. After explaining all the components of our solution, Section 4 presents the case study; the experimentation and the results obtained are discussed. Finally, Section 5 describes the conclusions that we have reached to after conducting the research and experimentation and considers possibilities for future work.

## 2. Related Work

The work presented in this paper deals with the benefits of monitoring, precise localization of home residents and real-time context-awareness, which offer possibilities for the development of systems and applications that foster energy efficiency. Those technologies also present a great potential for the development of Social Computing tools which would promote energy saving and good energy consumption habits. The main feature of this type of solutions is the collection of data through sensors [3]. This section of the paper outlines different proposals that are focused on the optimization of energy consumption in households through context-aware systems. Moreover, indoor Real Time Localization Systems used for energy saving solutions are analysed. Finally, the section identifies and analyses related works that employ Social Computing for efficient energy consumption.

### 2.1. Activity Monitoring and Contextual Information for Energy Efficiency

Nowadays, with the boom of Internet of Things (IoT) solutions, context-aware systems have become more commonly implemented in our surroundings, which is due to their reduced cost and ease of use and integration [13]. Furthermore, sensor networks are widely used to collect environmental parameters in homes; they are a source of information that supports the decision-making process [31] and, in particular, aids energy optimization and learning [20].

In the literature, we find multiple works that use sensors to contextualize environments for the purpose of saving energy. Temperature, lighting and electrical consumption monitoring systems are the most commonly used. Among them, we find the system proposed by Thomas et al. which determines activity in homes by integrating motion, door, lighting and temperature sensors [15]. Additionally, their system allows customizing services depending on the detected behaviour patterns. Moreover, Hong et al. use presence, lighting and window status sensors in their work to model residents’ behaviour, demonstrating that they are able to decrease energy consumption by up to one third [3]. A simpler work conducted by Asif et al. analyses the importance of lighting control and the presence of users for energy saving at homes [32]. We can also find commercial products such as nest Thermostat [33], a smart thermostat designed by Google that allows managing house temperature remotely. Thanks to its connection with the smartphone, the system is also able to determine the number of people in the house, to stop the heating system, calculate the duration of a fixed temperature in the home, or register the users’ favourite temperatures. To promote energy saving, it provides feedback to users and offers a rewards system (leaf reward) to encourage more responsible energy use. However, the solution still has some limitations: it only takes into account the main user, does not generate recommendations on consumption, does not learn from the behaviour of users, and does not integrate social functionalities. Another approach, presented by Morales et al., uses a complex device to monitor, control and manage electrical loads in homes to favour energy saving [14].

In relation to the monitoring of activities, different criteria have been used when dealing with this problem. Nguyen performs an important classification in his work [4] which identifies the most relevant information to be monitored: occupation, preferences, prediction of occupation and detailed activities. Real-time occupation in rooms is the information that is taken as the basis of multiple works. Thus, in [34], the lighting is controlled according to occupancy; in [35], temperature is automatically reduced when there is no occupancy; and, in [36], both are combined, controlling lighting and air conditioning depending on the occupancy of meeting rooms. The inclusion of users’ preferences enriches the systems that use occupancy to improve energy efficiency. Thus, some systems use timers to turn off lighting when the last movement has been detected [4]. Other systems, such as [37], follow users to find a balance between lighting preferences and minimization of electricity consumption. Grouping more preferences, in [38], users are tracked and their preferences for lighting, temperature and ventilation are collected.

Predictive models are resources that aim to optimize the efficiency of the actions taken [4]. This reduces the response time when automating actions, such as changing the temperature or turning off lights. There are multiple works in this area, such as ACHE [39], which predicts occupancy with hours in advance, entrance areas or hot water consumption. Other works, such as [40], predict the hours of entry and exit from home. Finally, works such as [41] combine occupancy prediction with weather forecasting to optimize temperature.

Finally, the activities that usually take place in the homes are a good guide for optimizing energy consumption (watching TV, cooking, use of washing machine, etc.). In this sense, in [42], three activities are identified (sleeping, working or leisure) to determine their needs and to adjust temperature and lighting. On the other hand, Nguyen and Aiello [43] recognize typical activities in the offices and Kim et al. [44] identify who is in an area of interest within the house and what activity they are doing, limiting these activities to three (watching television, using the coffee machine or using a lamp).

The analysis of these works helps to identify three main parameters that most influence energy consumption in homes: temperature, lighting and plug load [4]. Furthermore, the studies show that context-aware information favours the procurement of consumption patterns, as well as decision making, enhancing energy saving. In addition, the literature approaches activity monitoring with interesting criteria, such as the prior identification of activities in which energy savings could be reached. However, most works only postulate the use of these systems, without actually applying them to real environments. Moreover, there is a lack of solutions that combine localization and tracking of users with the collection of context-aware data, especially those that use the same communication platform to carry out both tasks. Some of the solutions that include activity monitoring are limited to few predefined activities. The following section focuses on the benefits that real-time localization provides and analyses several works that use these systems to improve energy savings.

Conclusively, the use of intelligent techniques and the connection between different systems and users are not addressed in these works. The Social Computing paradigm offers resources and techniques that support the active participation of both users and the deployed systems in data collection. These techniques allow for the prediction, adaptation and anticipation of users’ actions, improving the energy saving system. Section 2.3 addresses the use of Social Computing in energy saving systems, and explains the advantages it has in encouraging the acquisition of good energy practices in households.

### 2.2. Real Time Localization Systems and Energy Saving

As stated in many studies, household occupancy is an important parameter that should be measured when trying to understand and identify the parameters that have an influence on energy consumption in homes [4,21], even when unoccupied [45]. Thus, indoor localization systems play an important role in the proposed solutions. In this section, we present and analyse different types of indoor localization systems that have been used in previous works to approach this issue.

Among the most commonly used technologies for detecting users in home are motion sensors. Among which, PIR (*Passive InfraRed*) sensors are the most extended thanks to their ease of use and low cost. Some solutions, such as the one presented by Lee et al. in [46], uses this technology to detect residents in homes and monitor their presence. Some solutions aim to develop more accurate tracking, by including more elaborated detection algorithms in which household components, such as furniture, are included to obtain precise user location [47]. On the other hand, recent studies, such as that presented by Moreno et al., combine this type of sensors with RFID (*Radio Frequency IDentification*) systems to control different devices and improve localization [16]. In other cases, as in [48], door sensors and PIR sensors are used to detect occupancy in homes and to predict temperature control strategies. These solutions are generally good at detecting occupancy in rooms, they are easy to deploy and their binary operation facilitates data analysis. However, they present a high number of false positives and their localization accuracy is poor [49]. In addition, a parallel system is required for data transmission, storage and analysis.

The detection of occupation in buildings has also been approached through the use of video cameras. For example, Bemezeth et al. devised a system for tracking users through video cameras, taking into account points of interest, which can be used in energy-saving solutions [50]. This type of systems is a logical solution for buildings in which there is an existing CCTV (*Closed-Circuit TeleVision*) system. However, it presents serious privacy problems. In addition, its implementation in homes supposes a very high price, both at economical and computational level.

To improve the accuracy of localization systems, some solutions use active RFID technologies to monitor residents. In these cases, users must carry a tag that allows them to be identified and located at any time. The SPOTLIGHT project prototypes a system that uses this technology to determine how close the users are situated to electrical appliances and thus determines the consumed energy depending on their position [44]. Wi-Fi networks are postulated to be great helpers when it comes to detecting occupation in buildings and homes. Consequently, the Sentinel project uses these networks in public buildings to determine their level of occupancy, achieving energy savings of up to 17.8% [51]. Other fingerprinting techniques require complex calibration processes and do not allow combining sensing and localization systems with a single deployed infrastructure [8,52]. In other works, localization accuracy is calculated using the measurements of the RSS (*Received Signal Strength*) and TOA (*Time Of Arrival*) received from the WLAN devices [53].

Recent trends use localization systems based on BLE (*Bluetooth Low Energy*) technology to locate users. Their installation costs are low and the system can integrate simple actions, such as the switching on and off the thermostats and lights [18]. Systems based on this technology require a slightly dense anchor infrastructure in the area in which they perform; this density depends on the required accuracy. Furthermore, these technologies are starting to be integrated with different mobile technologies [54], as shown in the project [55], which uses iBeacons and Android systems to track users and achieve a 10% accuracy improvement and 15% of energy savings. Generally, all of them provide a very limited localization accuracy, offering in the best case, the use of active technologies like active RFID or BLE, which can find the position of users by determining the anchor that they are the closest to, these systems can generate a high number of false positives in small environments such as houses.

Finally, navigation systems based on inertial sensors are discarded since the drift problem causes them to be inaccurate [56].

To summarize, there is a lack of works that combine localization and contextual information in an efficient way, for the development of solutions that favour energy saving behaviour at home. CAFCLA combines sensors and real-time localization transparently, without the need for any calibration when deploying this single infrastructure, which provides a high level of accuracy and fast deployment [57].

### 2.3. Social Computing for Energy Efficiency

The emergence of the Social computing paradigm enhanced collaboration between humans and machines, solving social problems by using innovative sociotechnical tools [26]. Virtual organizations (VOs) of agents are postulated to be one of the most powerful tools for their support [58]. Some of the functionalities that make them a strong candidate, are their ability to control agent behaviours by the inclusion of normative regulations, their dynamism when forming agent groups and when managing the entry and exit of components, and their ability to describe functional behaviours and structural compositions [59]. The application of Social Computing techniques leads to the creation of Social Machines, entities that are managed by both technical and social practices. Examples of such entities are the Twitter dynamics prediction system, which uses behavioural data [58], or the Amazon recommendation system [60].

Several solutions aim to promote energy saving by fostering consumers’ engagement in good energy consumption practices [25,61]. Within this topic, Zhou and Yang propose a framework focused on different research areas (energy, social and information) to understand the social issues that affect energy consumption, including demand-response and intervention strategies for efficient consumption [1]. Barrios-O’Neill argues [61] that consumer behaviour can be influenced by strategic social interactions and, in order to improve this area, proposes a Socially Dynamic Communications Framework to foster effective engagement in the designed interactions.

Despite all of the above, no working infrastructure integrates different technologies, communications protocols, intelligent management and multiple ways of social interactions. Furthermore, the use of context-awareness and, especially, of localization systems is not addressed in depth, weakening human-machine interactions.

## 3. CAFCLA Approach for Activity Monitoring and Energy Efficiency

From the analysis of the solutions presented above, we can reach to the conclusion that, contextual information, localization and Social Computing offer great potential for the development of energy saving systems. Consequently, this work makes use of CAFCLA [57], a framework based on localization, and context-awareness and which also integrates Social Computing tools. CAFCLA serves as a basis for the development of an intelligent recommendation system that encourages responsible energy consumption in households.

### 3.1. CAFCLA Description

**Figure 1:**
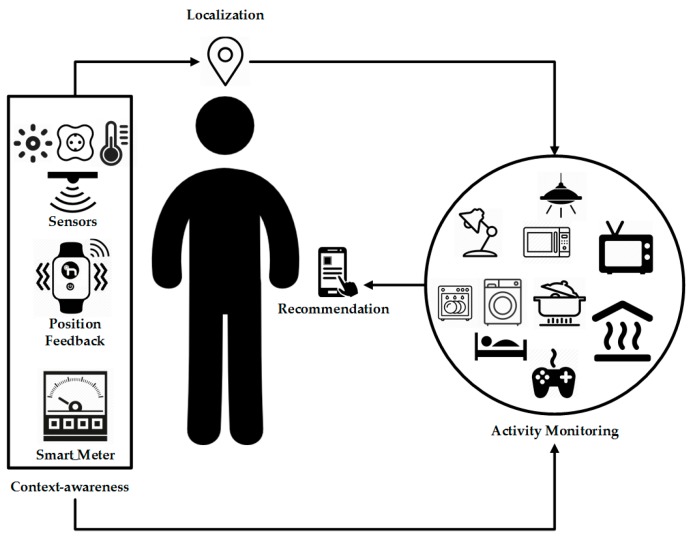
The presented model collects contextual information and users’ location. These data, merged with user activity monitoring, allow generating recommendations that foster energy saving.

The use of multiple technologies can help promote energy saving in households. The objective pursued in this work is to identify users’ activities in homes using Wireless Sensor Networks and a Real-Time Localization System for the collection of data. Once the activities are identified, thanks to the work done by a social machine that is responsible for data processing and analysis, a system of recommendations makes use of the data collected by the sensors, the localization system, the electricity consumption and the activities identified, to promote efficient energy usage among users. Figure 1 shows all the components involved in the system. The implementation of this type of solutions helps users to make a more efficient use of energy in their homes. Additionally, it helps to identify users’ behaviour when consuming energy; these data can be used to research and develop solutions that allow us to save energy.

Although CAFCLA was conceived as a framework for the development of collaborative learning activities [57], its universality allows it to be applied to multiple domains, in both academic and non-academic environments. This is the case of the scenario shown in Figure 1, in which the framework is used to foster and enhance the acquisition of good energy habits at home using a recommendation system. Despite not following a regulated learning process, by encouraging such habits, it creates a process that could be considered educational.

The best aspects of CAFCLA are its transparency and ease of use. This is because these technologies can be used at any time without users having to worry about the difficulties that their integration entails. CAFCLA has been developed in C++ and supports the integration of different communication technologies and devices. Moreover, CAFCLA makes it easy to develop and deploy solutions such as the API, its offers can be accessed using Java, C# or .NET.

The biggest novelty of CAFCLA lies in the integration and combination of the following functionalities within the same structure:

- It obtains contextual information through the implementation of Wireless Sensor Networks which collect data from multiple sources to define the environment. This includes sensors that collect environmental parameters (temperature, humidity or lighting) or data on device usage (the switching of lights and even the status of blinds and windows).
- It implements a Real-Time Localization System that allows users to be identified and tracked at all times. Users’ positions permit identifying patterns of behaviour that help to describe good or bad energy consumption habits.
- It integrates a social machine that provides users with recommendations. The social machine uses virtual organizations of agents to provide the system with intelligence. Monitoring of all contextual parameters, localization and tracking of users, management of communications and data, as well as the generation of recommendations for users encourage the efficient use of energy.

**Figure 2:**
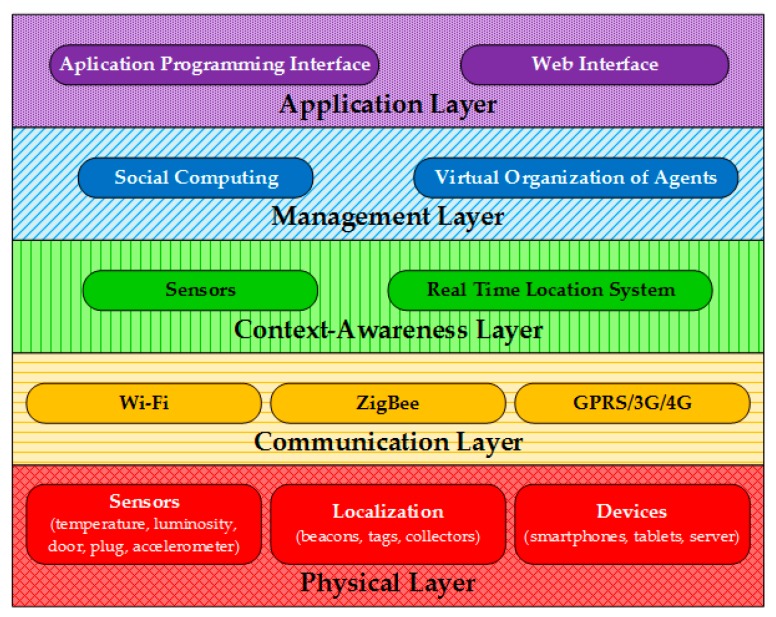
CAFCLA (Context-Aware Framework for Collaborative Learning Applications) layers diagram and the devices and technologies implemented within each of them for this use case.

**Figure 3:**
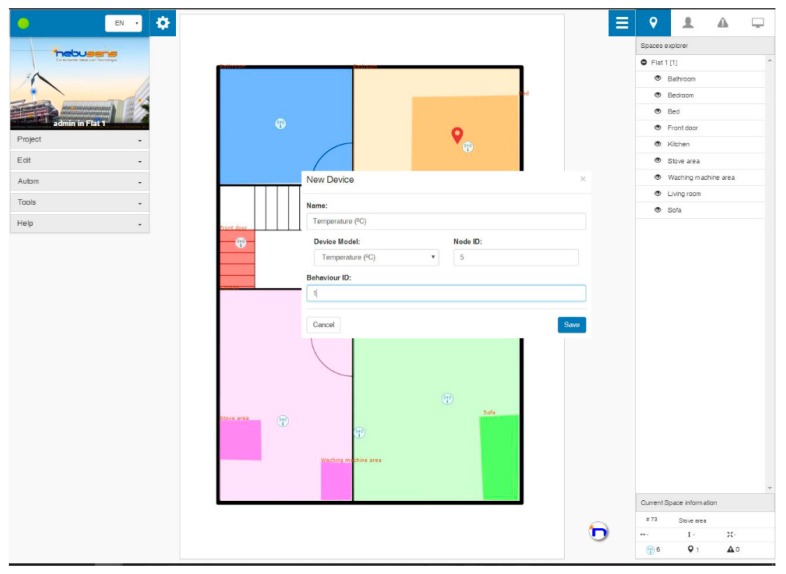
CAFCLA implements tools, which, among other functions, define the areas of activity identification and configure sensors.

The CAFCLA design follows a scheme of layers, each layer offers a set technologies and tools needed to meet the functionalities of the system, as shown in Figure 2:

- *Physical layer:* It is made up of all the devices and infrastructure used by CAFCLA. They can be classified in three different categories:
  ○ *Sensors:* The infrastructure that collects all the information needed to characterize the environment, including temperature, lighting, door/window and electricity consumption plug sensors. It also integrates an accelerometer within the localization tag to better identify the level of users’ activity. The wireless sensor network sends all the different physical measures gathered or the consumption of the monitored electrical points through the network.
  ○ *Localization*: The process of locating and tracking users requires a set of beacons (anchors) within the house and tags (targets) that identify each user individually. The target sends information with different parameters to the beacons. Similarly, the beacons resend it to the collectors, which, in turn, send it to the server where the parameters are processed and the precise position of users is determined, as well as the level of their activity.
  ○ *Devices*: Devices are all the equipment needed by the system that does not produce context-aware or localization data. This includes mobile devices such as smartphones or tablets which serve as an interface and are used to provide users with recommendations. In addition, the server that is deployed to store data and run the management system, including the localization engine and the social machine. Finally, it includes a protocol converter which acts as an intermediary between the sensor and localization infrastructure and the Internet.
- *Communication layer:* The CAFCLA design allows for the integration of any communication protocol that may be needed. In this case, the framework implements three communication protocols. The converters always implement two communication protocols. At one end, the ZigBee protocol (IEEE 802.15.4) which transmits signals and information to communicate with the localization and sensor infrastructure, and, on the other end, Wi-Fi or GPRS/3G/4G to send and receive data from the server. Finally, mobile devices receive and send information via Wi-Fi or GPRS/3G/4G protocol.
- *Context-aware layer:* Thanks to the use of CAFCLA and the integrated n-Core platform [33], both the wireless sensor network and the Real-Time Localization System can be deployed using the same physical and logical infrastructure; this simplifies the development of the Context-aware Layer. The n-Core platform consists of a set of hardware devices (RadIOn as beacons (anchors), IOn-E devices as sensors and Quantum v2 as tags (targets), all of them from Nebusens, Salamanca, Spain) and software tools that integrate the ZigBee communication protocol. These devices form a mesh network whose devices collect both contextual information, through sensors, and localization information, through beacons and tags. These data feed the recommendation system presented in Section 4. In addition, one of the major benefits of the platform is the duality of sensor nodes, as they can also act as localization anchors, which reduces the infrastructure of devices for the integration of any use case. Section 3.2 and Section 3.3 provide all the details on the RTLS integrated by CAFCLA.
- *Management layer:* This layer provides the social, logical and intelligence aspects of the framework. One of its main tasks is to classify the contextual information and correlate this information with the monitored activities. Following the methodology of previous works, such as [42,43,44], eleven recommendations have been predefined to be delivered according to the identification of the users’ activities. The recommendations that have been predefined within this development are as follows:
  - Turn off heating if the temperature is over 18 °C.
  - Turn off lights if lighting is over 200 lux.
  - Turn off heating and lights when the last person leaves the house.
  - Turn off lights if there is no movement in certain areas, for example when sitting on the couch watching TV, playing, etc.
  - Turn off room lights with no occupancy.
  - Turn off heating if no movement is detected at night and the temperature is above 18 °C.
  - Optimize the use of the heating schedule by identifying the times in which there are people at home.
  - Reduce the use of the washing machine by recommending an appropriate load and a low energy schedule.
  - Let us cook together: suggest cooking schedules or joint meals to users.
  - Organization of the bathroom: suggest a planned and serialized use of the bathroom to take advantage of the heat and the production of hot water.
  - Warn about stand-by consumptions in devices (televisions, consoles, computers, etc.).
- *Application layer*: This layer includes tools which configure all the sensors and define the areas of interest, such as beds (to identify when users are sleeping), couch (watching TV, reading, etc.), work areas (washing machine, cooker, etc.), front door, etc. Further, these tools also include mechanisms that define the activities to be identified or the conditions in which a recommendation should be triggered. Figure 3 shows the interface of the context-aware and localization systems, including the menu where new sensors in the system are registered.

The information collected by sensors, in particular by smart plugs, permits recognizing and classifying the activities performed by users. CAFCLA monitors temperature, lighting, electric consumption and users’ movement. Thus, according to the level of lighting in a room, the temperature, the electric consumption of any of the monitored devices (washing machine, dishwasher, TV or PC) and the hour and day of the week, the system will be able to identify different user activities such as watching TV, having breakfast or using the bathroom. The RTLS is a perfect complement to the data collected by sensors [52] since it provides the location of users and their activity level, enabling to infer for example, who is watching the TV or putting on the washing machine. In this respect, CAFCLA integrates an RTLS with an accuracy of up to 1 m and provides tools which, among other functions, define areas, place sensors, register users and define rules in order to generate and filter the information that is then processed intelligently, as explained in Section 4.

Having depicted the structure of the CAFCLA framework, the following section focuses on the techniques used to optimize localization accuracy and details the integration and performance of the RTLS within CAFCLA. Thus, the combination of localization and context awareness under a single infrastructure provides added value to the systems that address this topic.

### 3.2. Real-Time Localization via Multilayer Perceptron

The main objective for the implemented RTLS is to obtain the position of a target, $x_{t\in \mathbb{N}}\in {\mathbb{R}}^{2}$, on each frame of time, t∈ℕ, by using measurements given by the RSSI (*Received Signal Strength Indicator*), $z_{t\in \mathbb{N}}=r_{t\in \mathbb{N}}\in {\mathbb{R}}^{N_{t}}$, where $N_{t}$ is the number of anchors with known positions, $p_{i\in 1,2,\ldots ,N_{t}}$, that transmit to the target at time t.

To reach this objective, the process is divided in two steps:

- *Distance estimation:* During the first phase, the distance between the target and each anchor, $\|x_{t}-p_{i}\|{}_{i\in 1,2,\ldots ,N_{t}}\in \mathbb{R}$, is obtained by using real-time RSSI values, $z_{t\in \mathbb{N}}$.
- *Position estimation:* During second phase, the position of the target, $x_{t\in \mathbb{N}}$, is obtained by using the distance estimates, $p_{i\in 1,2,\ldots ,N_{t}}\in {\mathbb{R}}^{N_{t}}$, makes use of $d\left(N_{i,t}\right)$ and the position of each beacon within the environment $x\left(N_{i}\right)\;$.

**Figure 4:**
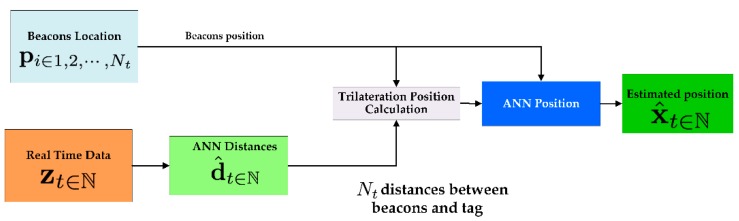
Architecture of the Multi-Layer Perceptron Neural Network used to calculate the distance between the anchors and the target within the system.

**Figure 5:**
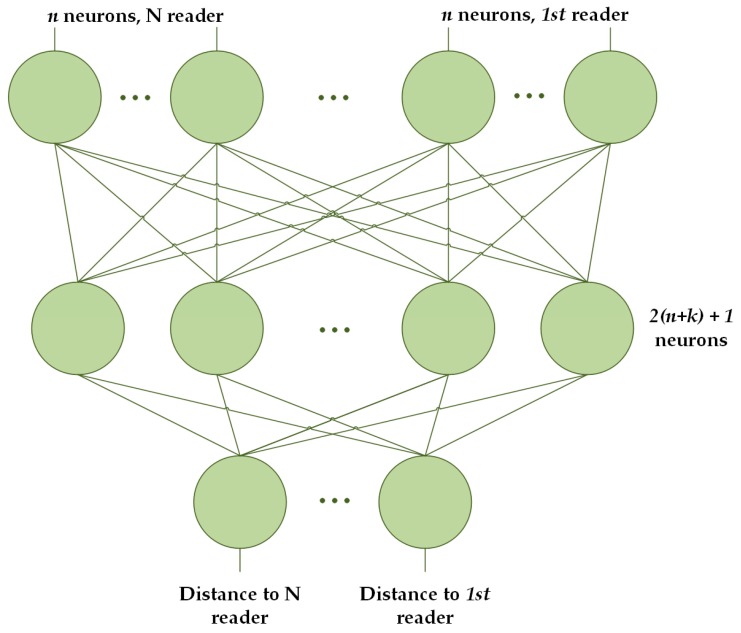
A schematic diagram of the two paths that follow the training and the real-time data: the calculation of the distances between anchors and targets and the calculation of the final estimated position.

In this work, target and anchors are responsible for sending the information needed to calculate the position of the first one, $x_{t}$, within the *Physical* and *Context-awareness Layers*. To calculate its position, the target sends information related to the RSSI of the signal, $z_{t}$. This information is sent every second if the user is in motion, being determined this state by a positive value of the accelerometer. These data, once on the server, are processed by the localization engine which estimates the position of the tag in the mentioned two phases:

- *Distance estimation:* During the first phase, the engine calculates the most probable distance between the target and the anchors, ${\hat{d}}_{t}={\left\|\hat{x_{t}-p_{i}}\right\|}_{i\in 1,2,\ldots ,N_{t}}$, based on the RSSI level, $z_{t}$. These values are calculated by using time series applied to ANN (*Artificial Neural Network*). ANNs permit for the use of time series, making it easier to forecast in situations where the estimation of the position from non-independent values with consecutive samples is not possible. Thus, the ANN is able to forecast a value according to the historical records. More concretely, an MLP (*MultiLayer Perceptron*) is used to provide a value according to the historical values [62]. Therefore, the neural network in this study is fed with both the current detected RSSI value and the RSSI values detected in previous time instants, $z_{t-l:t}$. The ANN has been trained considering several anchors at the same time to avoid the multipath effect. It is formed by (l+1) input neurons, with l the lag or number of previous times recorded. The intermediate layer of the Neural Network uses 2(l+1)+1 neurons and is configured following the *Kolmogorov Theorem* [63].
  Figure 4 shows that the ANN includes a number of input groups equal to the number of anchors considered in the estimation. Finally, the classification of the groups of input neurons is made attending to the current RSSI signal, with the first neuron corresponding to the reader that receives a higher RSSI and the last, the one that receives the lowest.
- *Position estimation:* The second phase calculates the position of the target, $x_{t}$, by using the distances calculated in the first phase, ${\hat{d}}_{t}$, and the beacons’ fixed location within the environment, $p_{i\in 1,2,\ldots ,N_{t}}$. The distance estimates and anchors’ positions are introduced in the second ANN (MLP) that estimates the target’s position from the input data [62,64]. This ANN has been trained by an *error back propagation algorithm* with positions obtained by the application of a trilateration algorithm to the distance estimates. This algorithm calculates the maximum likelihood (ML) of adaptive distributions based on kernel mixtures [53]:
  (1) $${\hat{x}}_{t}=\arg\underset{x_{t}}{\max}p\left({\hat{d}}_{t}|x_{t}\right)=\arg\underset{x_{t}}{\max}\overset{N_{t}}{\underset{i=1}{\prod }}p\left({\hat{d}}_{i,t}|x_{t}\right)$$
  where $p\left({\hat{d}}_{i,t}|x_{t}\right)$ is the likelihood function of the target’s position for the distance estimate with respect to the ith anchor where, for one kernel:
  (2) $$p\left({\hat{d}}_{i,t}|x_{t}\right)\approx \frac{1}{h}K\left(\frac{\|x_{t}-p_{i}\|-{\hat{d}}_{i,t}}{h}\right)$$
  where K(·) is a kernel function with bandwidth h [65].
  For the trilateration algorithm, we use a Gaussian kernel due to the tractability constraints of the ML. However, by using the ANN, we avoid the high processing times required by other kernels, by dealing with the complexity vs. accuracy trade-off. The number of neurons of the ANN is n in the input layer, 2n+1 in the hidden layer, and 1 in the output layer.
  Figure 5 shows how the problem is resolved in a schematic way. This model will provide the position of the tags within the localization environment, at every second.

### 3.3. Integration within CAFCLA and Performance of the RTLS

In this section, we assess the performance of the localization system in comparison with conventional techniques. Note that the devices have been designed by the authors for the purpose of localization and the results obtained with novel techniques will outperform those obtained with conventional, off-the-shelf devices.

As introduced in Section 3.1, CAFCLA integrates the functionalities of an RTLS based in the ZigBee communication protocol. The deployment of the integrated localization system requires a number of beacons (anchors) to be distributed in the scenario, directly proportional to the required accuracy. Those beacons are Sirius RadIOn devices, part of the *Physical Layer* of the framework. They transmit in the 2.4 GHz frequency band with a transmission power of 3 dBm and patch antennas. In addition to placing and locating each of the anchors in the houses, the software allows determining the different areas within the house with high granularity, as they are not only defined at the room level, but more precisely such as the stove area, the washing machine, the sofa and the armchairs, the different tables, the beds and the front door. This information is very useful for identifying the activity that the user is performing at a given moment and, thus, provide a recommendation accordingly.

The target carries a mobile tag (Quantum v2, also part of the *Physical Layer*) with an integrated accelerometer, a vibrator to receive warnings and a button that allows sending feedback on the recommendations sent by the system. This tag transmits a set of signals to the beacons with their RSSI values. All the information acquired by the devices in the *Physical Layer* is transported via ZigBee, in the communication between readers, and Wi-Fi or 3G/4G from each house to the server. Within the server, all the functionalities that make up the *Context-Awareness*, *Management* and *Application Layers* are running to perform all the services offered by CAFCLA. Within the *Context-Awareness Layer*, the received RSSI signals are organized to serve as input data for the localization model within the *Management Layer.* Here, the position of each user within the house is estimated by means of the described localization model.

**Figure 6:**
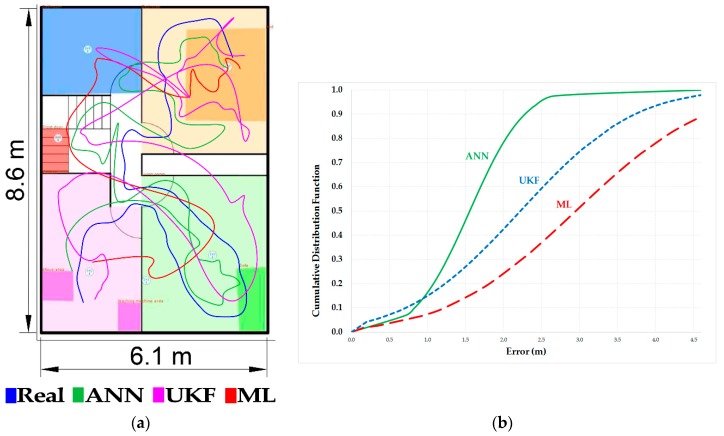
The presented localization algorithm remarkably outperforms conventional localization techniques based on UKF and ML. (**a**) The real route and the detected by the different techniques. (**b**) The error obtained with each technique regarding the real route.

Figure 6 shows the result of a case study where a target is localized on the basis of signals transmitted to six anchors every second. We compare the results with conventional techniques based on ML and Bayesian filtering. We call:

- ANN: The proposed localization approach based on the two MLP stages.
- UKF: A conventional Unscented Kalman Filter (UKF) based on the likelihood shown in Equation (2) [66].
- ML: A trilateration algorithm based on the likelihood function shown in Equation (2) [53].

As Figure 6 points out, the proposed two-step ANN obtains lower errors than conventional techniques, obtaining a RMSE (*Root Mean Squared Error*) of 1.66 m for the ANN, 2.53 m for the UKF and 3.26 m for the ML approach. This level of accuracy is highly useful for determining the position of each user inside the house, identifying when they are sitting on the sofa, cooking or at the front door ready to leave.

The next section addresses the use of Social Computing in energy saving systems, the development of the designed recommendation system, and the inclusion of Social Computing and virtual organizations of agents as cornerstones of intelligent and efficient management in the system.

### 3.4. Energy Efficiency Based on Social Computing

The great utility of localization and sensing data for the monitoring of users’ activities in their households requires a system that is able to manage this information efficiently and intelligently. As the title of this section suggest, Social Computing is postulated as a paradigm that maximizes the functionality of this information and allows simplifying and improving relationships between machines and humans.

The development of a recommendation system such as the one presented in this work requires a set of functionalities which are difficult to merge, as shown in the following.

The main objective of the recommendation system is to achieve a substantial reduction in energy consumption in households and improve the demand response through the promotion and acquisition of energy-efficient habits.

This work is based on three technological pillars: first, a precise localization system that allows to monitor users and their level of activity at all times; second, a scalable sensor network that gathers environmental parameters to characterize the environment in real-time; and, third, a social machine that provides intelligence to the system. CAFCLA allows implementing intelligence techniques through the *Management Layer*, which is responsible for covering all the social, logical and intelligence needs of the system. For this reason, this layer implements the VO-based social machine that supports the recommendation system. The implementation of the VO has been carried out using the JADE platform and the Jadex tool [67], an extension that provides a BDI (*Belief-Desire-Intention*) architecture to the JADE agents. Thus, Jadex agents work with concepts such as beliefs, goals and plans. Jadex has the advantage of allowing the programmer to introduce their own deliberative mechanisms. The platform allows implementing open multi-agent systems easily using different tools to create, manage and control virtual organizations, including organizational aspects.

**Figure 7:**
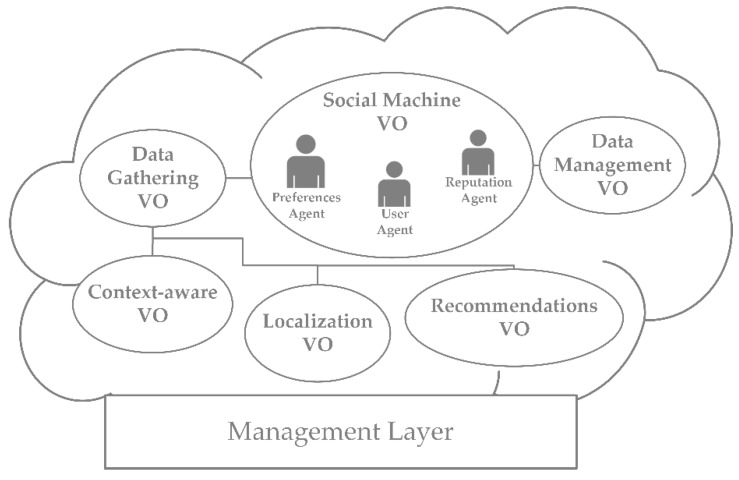
The social machine is supported by the VOs’ that manage the localization and tracking of users, the contextual information and the generation of recommendations which help foster good energy saving habits.

Figure 7 shows the different VOs included in the designed architecture:

- *Localization VO*: It collects the RSSI information and implements the localization algorithm. It is coordinated with de *Data management VO* to update the position of each user in real-time.
- *Context-aware VO*: It manages all the information gathered by the sensors. It is coordinated with the *Data management VO* to update the information collected by the sensors.
- *Data gathering VO*: It is in charge of the management of the data sources and the control of heterogeneous systems such as the localization system, the WSN and other data sources.
- *Data management VO:* It includes data reception, classification, storage and delivery. It classifies users’ preferences, identifies habits and patterns or the correlation among context-awareness and predefined activities, among others.
- *Social machine VO*: It manages the interaction among all the agents of the system and the social information extracted from them. The agents implemented in this VO are as follows:
  ○ *User agent*: It is responsible for user-user and user-machine interactions. They register all the information that is relevant to the recommendation system.
  ○ *Preferences agent*: It analyses the information stored by the data management layer and identifies and classifies energy preferences for each user and their energy saving habits.
  ○ *Reputation agent*: It manages the reputation of energy-efficient actions. This reputation is calculated using a Bayesian system that takes binary ratings as inputs [68]: when a recommendation is sent and accepted by the user, if it implies energy savings, then positive rating increases by 1 unit (α) and, if it does not imply energy savings, negative rating increases by 1 unit (β). The reputation $E\left\{p_{i}\right\}$ of the ith recommendation is calculated using beta probability density function (pdf) and its score is represented by the expected value of the beta pdf. Thus, the beta pdf is expressed using the gamma function, Γ(·), as:
    (3) $$f\left(p_{i};\alpha ,\beta \right)=\frac{\Gamma \left(\alpha +\beta \right)}{\Gamma \left(\alpha \right)\Gamma \left(\beta \right)}{p_{i}}^{\alpha -1}{\left(1-p_{i}\right)}^{\beta -1}$$
    $$\;s.t.\;0\leq p_{i}\leq 1\;\alpha ,\beta >0\;p_{i}\neq 0\;if\;\alpha <1\;p_{i}\neq 1\;if\;\beta <1$$
    Then, the expected value of the beta distribution is calculated as follows:
    (4) $$E\left\{p_{i}\right\}=\;\frac{\alpha }{\alpha +\beta }=\frac{1}{1+\frac{\beta }{\alpha }}$$
- *Recommendation VO:* It generates personalized recommendations which are sent to users to engage them in using energy more responsibly. It receives information from the reputation agent.

The next section describes the implementation of a case study in which the recommendation system is used to promote energy saving in households.

## 4. Case-Study: Recommendation System for the Enhancement of Energy Saving in Homes

This section describes the experimental set-up and the results of a case study designed to assess the performance of the presented framework for energy efficiency in households. First, it presents the recommendation system, its objectives and the recommendations that are provided to the users. Next, it shows the consumption results before and after the implementation of the recommendation system.

### 4.1. Description of the Case-Study

The proposed system has been deployed in five houses with three different typologies: Type I is one one-bedroom flat with one user; Type II are two two-bedroom flats with two users; and Type III are two three-bedroom flats with three users. All the houses have a kitchen, a living room and a bathroom except the three-bedroom dwellings, which have two bathrooms. As a whole, 11 users participated in the experiment. In Figure 3, we can find an example of how the system was deployed in one of the flats.

Furthermore, the WSN consists of a temperature and a lighting sensor and is deployed in all the rooms in every flat; in the kitchen, the bathrooms, the living room and the front door area, which implies a total of five measurement points for Type I, six for Type II and ieght for Type III. These points are created by an IOn-E device [69] connected through a digital I2C port to a RadIOn communication device. The IOn-E device integrates a light sensor model TSL2561 (AMS, Stiria, Austria), whose measuring range is between 0.1 and 40.000 lux. The brightness measurement is sent every 60 s to the server. The IOn-E device also integrates a high-precision temperature sensor SHT25 (Sensirion, Staefa, Switzerland). This sensor is capable of recording measurements between −20 °C and 100 °C with an accuracy of ±0.1 °C. For the purpose of this work, the sensors send the temperature to the server when there is a variation of ±0.5 °C.

Electric consumption is gathered from smart plugs in the living rooms and the bedrooms of each of the flats, and in this way the use of devices such as TV, computers, lamps, etc., can be detected. We use Cloogy plugs [70] in the living room and bedrooms. They include an electrical consumption sensor, that registers consumption every 15 min, with an accuracy of ±1% ± 0.5 W, and ZigBee communication capabilities, which provide data on consumption. The data are collected by a crawler, which then accesses the web service to publish consumption measurements. Every day, a different crawler obtains the aggregated hourly consumption data of each household from the API of the DSO connected to the smart meter, to which all the users have access.

One RadIOn device per room and a Quantum device will act as anchors and target for the localization system, which enrich the system thanks to its great accuracy.

**Figure 8:**
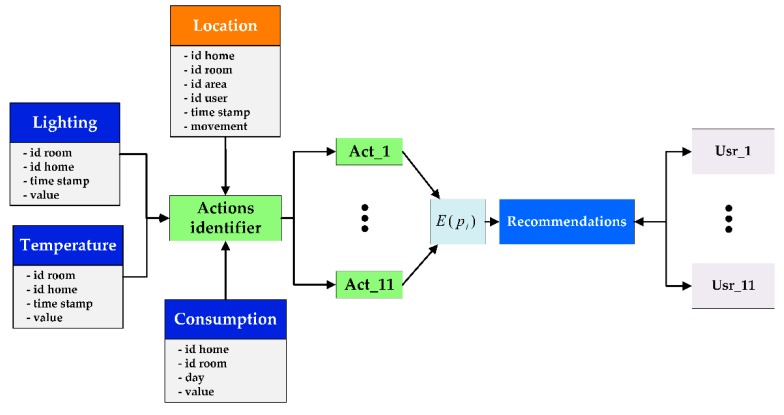
The social machine receives data from the localization engine and the sensor network to identify the actions that are being carried out. With the current and historical data gathered, the social machine calculates the reputation of each action to provide customized recommendations to the users.

The social machine implemented in this experiment receives real-time data from both the localization system and the WSN (see Figure 8). These data allow identifying patterns such as predefined actions, inefficient energy uses and potential improvements, by providing the users’ instantaneous situation and their surrounding environment. Moreover, the social machine calculates the reputation of each of the actions in order to recommend them to users. These recommendations are sent by email, they outline the benefits of a specific behaviour, the number of participants who have this energy saving habit and the improvements that these participants have achieved. Recommendations are fully and clearly explained, this aids rapid improvement. This recommendation process may not be taken into account by all users; some may not read the emails while others simply may ignore a particular behaviour recommendation. To help the system interpret users’ decisions, the localization tag carried by each user vibrated when they received a recommendation. If the user regards the recommendation as useful, they will press the tag button as a signal of positive feedback.

The next section describes the results obtained during the experimentation, and also compares baseline consumption data.

### 4.2. Experimental Results

The experiment was performed during two consecutive months. During the first month, the daily consumption data were collected for each flat to create a baseline consumption and compare it with the results of the experiment in the second month. The month of April was chosen for collecting baseline data and the month of May to perform the experiment, seeking to minimize the differences in weather conditions, pluviometry and sunlight that could affect the results, avoiding the months with extreme hot or cold. Moreover, these months were chosen because they are considered the most standard months in terms of work and daily life, which means that the participants were at home (and not on holidays).

To obtain the baseline data and to determine the recommendations, the deployed system collected data without sending recommendations for 30 days; the obtained results are shown in Table 1, where savings are calculated as follows:

(5) $$Savings=\frac{Baseline\;consumption-Reported\;consumption}{Baseline\;consumption}$$

As shown in Table 1, flat 1 displays significantly lower consumption during the development of the experiment in comparison to the monitoring phase in the first month, while the other flats experienced more moderate changes. Energy savings have oscillated between 13.78% and 21.98%. We can point out that the average energy savings per user were 17.08% (44.41 kWh) during the experiment.

**Figure 9:**
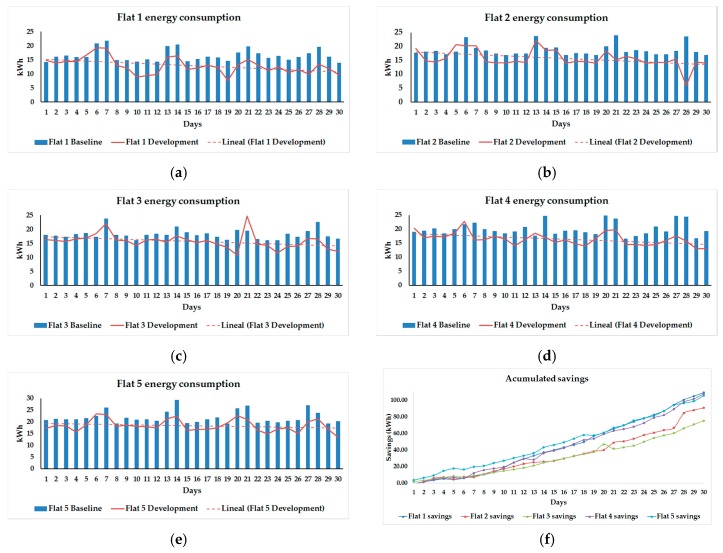
Daily energy consumption during the baseline and the experimentation periods in each flat (**a**–**e**); and accumulated savings (**f**). The results demonstrate the reduction in consumption and the lineal tendency shows how players acquire good habits along the experimentation.

Figure 9 shows daily consumption for each flat during the monitoring experimentation phases. The graphs show the levels of consumption for each day of the week. Levels increase during weekends, when users stay at home for longer and use the time to do the laundry, watch TV or cook. Furthermore, the negative tendency (dashed line) indicates that users are managing to save energy, thanks to the recommendations, and acquiring good energy consumption habits. Finally, the accumulated savings in Figure 9f asseverate that the use of the social machine is effective.

Table 2 shows the results of the Student’s *t*-test and Levene’s test. In this case, the difference between the reported average consumption during the monitoring and the experiment was significantly lower for the latter in all the cases, with a *p*-value of 0.000 in all cases for the mean differences, which demonstrates the effectiveness of the recommendation system in terms of energy savings and behavioural change.

The analysis of the results showed that the most frequent recommendations taken into account were those related to the use of heating (77%), lighting (84%) and switching off devices to avoid stand-by consumption (92%). However, recommendations on the optimization in bathroom usage (4%) or cooking at the same time (17%) did not have a high response, which shows that the social machine has operated more effectively in human-machine than in human-human relationships.

## 5. Conclusions

This paper presents a recommendation system that identifies users’ energy consumption behaviour patterns in their homes to promote more efficient energy usage. To achieve this aim, it uses the CAFCLA framework to integrate the infrastructure, a Real-Time Localization System and WSNs. The design of the system makes use of Virtual Organizations of agents that enabled for the development of a social machine which personalizes the recommendations sent to each user. Thanks to the precise traceability of each user, the system is able to encourage them to acquire good energy habits.

The conducted experiment involved the deployment of the required infrastructure in five houses with different typology and had 11 participants in total. The obtained results point out to the fact that the combined use of the localization system with the WSN and the social machine, allows determining the context that surrounds each user with high precision. The intelligent management of this information helps identify situations in which potential energy savings arise and, immediately, generate and send customized recommendations to encourage users to take energy saving actions.

More specifically, the proposed system attains an average energy savings of 17%, reaching savings up to 22%, while state of the art solutions achieved between 10% and 15%. Future work is addressed to validate this improvement and users’ adherence in the long term, including experiments with a control and experimentation group for one year. In addition, the proposed system provides a great flexibility by combining context-awareness, localization and Social Computing techniques. Multiple solutions have approached these problems independently but none of them has faced them together.

However, the accuracy of the system makes its implementation in homes very expensive for users. In this regard, it is necessary to improve the accuracy of localization using more standard devices, such as mobile phones. Moreover, although it is becoming easier to implement these solutions due to the boom of contextualization devices, future work will require the coordination of different stakeholders (promoters, developers, manufacturers, etc.) to benefit from the integration of technologies coming from different sources. Finally, with the aim of further decreasing energy consumption, future works will integrate techniques that consider demand response information and the price of energy.

## Figures and Tables

**Table 1:** Total consumption in kWh during the 30 days of baseline data collection, during the 30 days of the experiment, difference in the consumption and savings between the two periods.

Consumption	Flat 1	Flat 2	Flat 3	Flat 4	Flat 5
**Baseline (kWh)**	496.56	561.66	545.40	601.35	654.73
**Experiment (kWh)**	387.41	470.92	470.24	493.57	549.04
**Difference (kWh)**	109.15	90.74	75.16	107.78	105.68
**Savings (%)**	21.98%	16.15%	13.78%	17.92%	16.14%

**Table 2:** Results of the Student’s *t*-test and Levene’s test performed to assess the difference of means and variances between the baseline usage data and the data collected during the experimentation. All the flats present a lower percentage of energy usage after the experimentation.

	Baseline		Experiment					
Houses	Mean	Std.	Mean	Std.	t	*p*-Value (2-Tailed)	F	*p*-Value
**Flat 1 (kWh)**	16.553	2.179	12.914	2.838	5.569	0.000	1.449	0.234
**Flat 2 (kWh)**	18.721	2.140	15.696	3.065	4.431	0.000	2.006	0.162
**Flat 3 (kWh)**	18.179	1.761	15.674	2.733	4.219	0.000	1.853	0.179
**Flat 4 (kWh)**	20.045	2.363	16.451	2.234	6.052	0.000	0.162	0.689
**Flat 5 (kWh)**	21.823	2.661	18.301	2.550	5.232	0.000	0.010	0.920
